# Socio-Economic Status of Patients With Type 2 Diabetes and Hypertension Attending the Ahmadu Bello University Teaching Hospital, Zaria, North-West Nigeria

**DOI:** 10.5539/gjhs.v7n1p280

**Published:** 2014-10-09

**Authors:** Stanley Irobekhian Reuben Okoduwa, Ismaila Alhaji Umar, Sani Ibrahim, Fatima Bello, Uche Samuel Ndidi

**Affiliations:** 1 Department of Biochemistry, Ahmadu Bello University, Zaria 810001, Kaduna State, Nigeria; 2 Endocrinology Unit, Ahmadu Bello University Teaching Hospital, Zaria 810001, Kaduna State, Nigeria

**Keywords:** Type 2 diabetes, T2D, hypertension, HTN, lifestyle, socio-economic status

## Abstract

Hypertension (HTN) and Type 2 diabetes (T2D) are lifestyle interrelated diseases of global significance. Interestingly, the prevalence of these diseases in Africa and indeed Nigeria seems to be on the increase. This study, therefore, investigated the socioeconomic status (based on income, education and occupational activity) of 400 subjects (52% female and 48% male) aged 20 years and above who were sampled randomly among the newly diagnosed HTN and/or T2D cases at the Ahmadu Bello University Teaching Hospital, Zaria, North-West Nigeria. A semi-structured questionnaire was used to collect information from the subjects. From the result obtained, most of the respondents who live in towns or city suffer from either HTN or T2D while more town dwellers (28%) suffer from a combination of both diseases. It was also discovered that most respondents who suffer from HTN and from a combination of HTN and T2D belong to the old generation (60-79 years). There is higher prevalence rate of diabetes among the respondents who had no formal education or attended only basic Arabic schools. Most respondents who earn good income (₦50,000-₦100,000 and above ₦100,000) suffer HTN, T2D and a combination of both diseases. Those engaged in heavy occupational activities had the lowest prevalence of the disease compared with those of light or moderate occupational activities. These data will be found useful in planning intervention healthcare preventive programs especially on public enlightenment workshops and seminars to educate the populace on the importance of lifestyle modification, healthy diet and regular exercises.

## 1. Introduction

Hypertension (HTN) has become a major contributor to global disease burden. It has been ranked as one of the leading preventable causes of premature death worldwide especially in developing countries (Yang et al., 2012). Studies have projected up to 60% increase in the numbers of hypertensive adults by the year 2025 (Kearney, Whelton, Reynolds, Muntner, & Whelton, 2005). According to the report of the WHO, at least 7.8 million people are affected by HTN in Nigeria (Kadiri, Walker, Salako, & Akinkigbe, 1999) and 1.0 billion individuals across the globe (Whelton, 1994; He & Whelton, 1997). A 2012 report by Ogah et al. (2012) puts the figure of HTN sufferers in Nigeria at 22.5%, which is approximately 30 million Nigerians based on the census that put Nigerian population at about 160 million people. The global public health burden of HTN has indeed been suggested to be unequally spread across socioeconomic strata (Kearney et al., 2005).

Similarly, Type 2 diabetes (T2D) is increasing globally and it is the third leading fatal disorder after cancer and heart disease. The present estimate of the number of persons diagnosed with T2D as at 2013 is 382 million across the globe (International Diabetes Federation [IDF], 2013). The figure has been estimated to increase to about 592 million by the year 2035 as a result of increase in population growth, increased sedentary life style and dietary habit (IDF, 2013). In Nigeria about 3.9 million adults between the ages of 20 and 79 years are diagnosed with diabetes mellitus (Kadiri et al., 1999) and T2D, which is also called Type 2 diabetes mellitus account for about 90% of the cases (Chijioke, Adamu, & Makusidi, 2010). A much more recent report put the figure of T2D sufferers in Nigeria at 5.12% of the total population which is approximately 8.19 million (Chijioke et al., 2010). The public health burden of T2D has been reported to be unevenly distributed across socioeconomic strata (Dray-Spira, Gary-Webb, & Brancati, 2010).

HTN and T2D are interrelated non-infectious diseases that are chronic and of global significance (Ginter & Simko, 2012; Centers for Disease Control and Prevention [CDC], 2012). The lifestyle of an individual or group of people has been known to be a function of socioeconomic status (Ginter & Simko, 2012; Olah, Gaisano, & Hwang, 2013). The socioeconomic status (SES) of the general population may help explain why the epidemic is on the increase. It has been reported that the prevalence of HTN is 1.5 to 2 times greater in patients with T2D as compared to non-diabetic subjects (Basavegowda, Shankarappa, Channabasappa, Marulaiah, & Hathur, 2014). The socioeconomic variables that dictate the quality of individual’s life are income, education, and occupation, which have been shown to result in differential opportunities for maintaining quality health (Denis, Bellefontaine, Marganne, Somasse, & Drielsma, 2011). Socioeconomic stratification is, therefore, the key to understanding affordability of health services, amenities and purchasing capability (Kumar, Dudala, & Rao, 2013). When it is taken as a summation of education, occupation and income, it reflects the value system expected for that level of education, occupation and income (Kumar et al., 2013; Basavegowda et al., 2014). Access to healthcare is vital for good health, unfortunately, people of low SES encounter many barriers to obtaining quality healthcare (Olah et al., 2013).

The association between HTN and T2D has been a subject of debate over the last decade (IDF, 2013; American Diabetes Association [ADA], 2014; Okoduwa, Umar, Ibrahim, & Bello, 2013). Several studies from developing countries have shown either no relationship between SES in the association between HTN and T2D (Rajput, Rajput, Singh, & Bairwa, 2012). Some other studies have shown that high SES is linked to an increased risk of T2D (Corsi & Subramnian, 2012; Zhang et al., 2013). However, there are limited information on the role of SES in the association between HTN and T2D. Earlier studies have shown, howbeit, that there is higher mortality among people with both HTN and T2D who live in rural areas or of lower educational status than among people from urban areas or higher educational attainment, but none have described whether the strength of the association between HTN and T2D varied by SES (Dray-Spira et al., 2010; Rajput et al., 2012). Indeed, income inequality in the Nigerian state, the increasing numbers of low income earners, and the further widening of the gap between the rich and the poor make the research imperative.

There are few studies on socioeconomic discrepancy in HTN and T2D in Nigeria, with those available often limited by examining only one socioeconomic indicator or not adjusting for other important socio-demographic and health factors (Kadiri et al., 1999). This article addresses some of these limitations, as indicated by both educational attainment and average monthly income as each may offer better perceptive of the pathways that link SES to HTN and T2D.

## 2. Methodology

### 2.1 Study Design

This study was a cross-sectional descriptive survey using a semi-structured questionnaire. The questionnaire covered settlement, fruit and vegetable consumption, age, sex, income, educational attainment and occupational activity. The research assistants were trained in basic interviewing techniques. The questionnaire was pre-tested for flow of questions and for validity and it was conducted in Zaria, Kaduna state, North-West, Nigeria.

### 2.2 Data Source and Participants

A total of four hundred (400) subjects (52% female and 48% male) aged between 20 and 80 years were randomly selected for this study from the Ahmadu Bello University Teaching Hospital (ABUTH) Shika, Zaria and Ahmadu Bello University (ABU) Samaru Main Campus, Zaria, North-West Nigeria. The study was conducted between August 2012 and July 2013 and the subjects were divided into four groups of 100 persons each namely: Non-Diabetic Hypertensive Group (NDHG), Diabetic Normotensive Group (DNG), Diabetic Hypertensive Group (DHG) and the Non-Diabetic Normotensive Group (NDNG). All the subjects were examined physically through the assistance of medical experts at the hospital. All measurements were taken by the same person in order to avoid subjective error. The NDHG was established based on the diagnostic criteria established by the Joint National Committee on Prevention, Detection, Evaluation, and Treatment of High Blood Pressure, which is ≥140/90 mmHg (National Institutes of Health [NIH], 2004). The DNG was established based on the criteria of the World Health organization (1999). Fasting venous plasma glucose above 7.0 mmol/l and/or 2-hours post-prandial plasma glucose above 11.1 mM using Glucose Oxidase Method was adjudged diabetic (ADA, 2014; Gavin et al., 1998; Alberti & Zimmet, 1998).

### 2.3 Ethical Consideration

The purpose of the study was explained to all the subjects and a written informed consent was obtained from them. The study was ethically approved by the Ahmadu Bello University Teaching Hospital Ethical Committee and in accordance with the Helsinki Declaration.

### 2.4 Selection Criteria

Patients diagnosed with HTN and/or T2D on their first visit at the Ahmadu Bello University Teaching Hospital (ABUTH), Shika, Zaria were selected. The control subjects (NDNG) were apparently healthy non-diabetic/non-hypertensive volunteers matched for age, sex and body mass index with the study population. They were selected from the Ahmadu Bello University Teaching Hospital Shika and the Ahmadu Bello University, Main Campus, Samaru, Zaria, North-West Nigeria.

Patients who are present or past smokers, patients who take alcohol, patients who have hepatic disease or take lipid lowering drugs were excluded. Also were excluded are patients who are on antioxidant vitamin supplements, probucol, allopurinol, quinidine, disopyramide, or other drugs that affect serum lipid peroxidation and antioxidant values. In addition, patients who are <20 or >79 years of age or fail to give a written consent were all excluded.

### 2.5 Statistical Analysis

All statistical analyses were done using simple descriptive analysis and SPSS software program (IBM SPSS v.19 Inc., Chicago Il, USA).

## 3. Result

Table 1 shows the prevalence of T2D and/or HTN by settlement, and by fruits and vegetable consumption. From the result, it was discovered that most of the respondents who suffer from HTN or T2D live in the town or city while most who suffer from both T2D and HTN are town dwellers (28%) followed by rural dwellers. Most of the respondents who rarely eat fruits and vegetable suffer from HTN, T2D and a combination of both compared to the respondents who consume fruits and vegetables.

**Table 1:** Prevalence of Type 2 diabetes and/or Hypertension by Settlement, and Fruits and Vegetable Consumption among all the Respondents

VARIABLE	Frequency	Population (%)			

		NDNG	DNG	NDHG	DHG
Total	400				
SETTLEMENT					
Village	60	40.0	26.7	20.0	13.3
Town	164	22.0	28.0	23.2	26.8
City	176	22.7	21.6	28.4	27.3
FRUITS AND VEGETABLES					
Daily	132	45.5	16.7	21.2	16.7
Weekly	148	20.3	35.1	20.3	24.3
Rarely	168	6.0	44.0	25.0	25.0

DNG:- Diabetic Normotensive Group; NDHG:- Non-Diabetic Hypertensive Group; DHG:- Diabetic Hypertensive Group; NDNG:- Non-Diabetic Normotensive Group.

The prevalence of T2D and/or HTN by sex and age is presented in Table 2. It was discovered that more females than males suffer from T2D while slightly more males suffer a combination of both diabetes and HTN. The ratio of male to female who suffer from HTN was 50:50. In relation to age, it was discovered that most respondents who suffer from HTN and those who suffer a combination of HTN and T2D are in the old generation (60-79). However, the middle aged respondents suffer most from T2D compared to the young and old generations.

**Table 2:** Prevalence of Type 2 diabetes and/or Hypertension by Sex and Age in all the Respondents

Group	Population (%)			

	NDNG	DNG	NDHG	DHG
Sex				
Male	26	22.9	25	26
Female	24	26.9	25	24
Age				
Young age (20 – 39)	43.4	24.5	13.2	18.9
Middle age (40 – 59)	18.9	31.6	26.3	23.2
Old age (60 – 79)	17.3	13.5	34.6	34.6

DNG: Diabetic Normotensive Group; NDHG: Non-Diabetic Hypertensive Group; DHG: Diabetic Hypertensive Group; NDNG: Non-Diabetic Normotensive Group.

**Figure 1:**
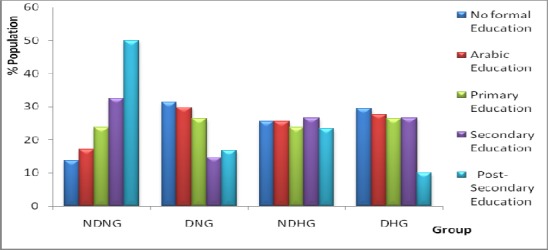
Prevalence of Type 2 diabetes and/or Hypertension by Level of Education among Respondents DNG: Diabetic Normotensive Group; NDHG: Non-Diabetic Hypertensive Group; DHG: Diabetic Hypertensive Group; NDNG: Non-Diabetic Normotensive Group.

Figure 1 shows the prevalence of T2D and/or HTN by level of education. It was discovered that most respondents who have T2D had either no formal education or attended only basic Arabic school while the respondents who have post-secondary education are the least sufferers of HTN and T2D. On the average, level of education does not seem to have effect on the respondents who suffer from HTN.

**Figure 2:**
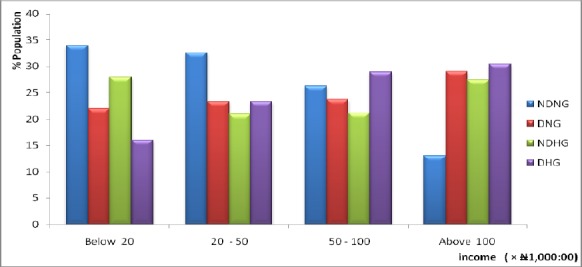
Prevalence of Type 2 diabetes and/or Hypertension by Average Monthly Income of Respondents DNG: Diabetic Normotensive Group; NDHG: Non-Diabetic Hypertensive Group; DHG: Diabetic Hypertensive Group; NDNG: Non-Diabetic Normotensive Group

The prevalence of T2D and/or HTN by average monthly income is presented in Figure 2. It was discovered that most T2D, hypertensive and sufferers of both diseased condition are high income earners, i.e. people who earn between ₦50,000 – ₦100,000 and those who earn above ₦100,000.

**Figure 3:**
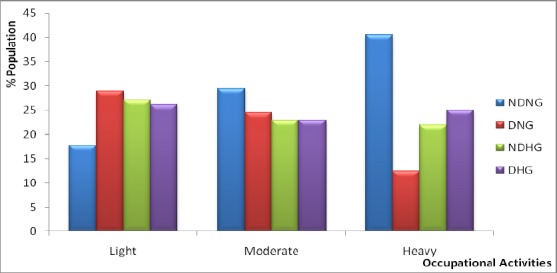
Prevalence of Type 2 diabetes and/or Hypertension by Occupational activities of Respondents DNG: Diabetic Normotensive Group; NDHG: Non-Diabetic Hypertensive Group; DHG: Diabetic Hypertensive Group; NDNG: Non-Diabetic Normotensive Group.

Figure 3 shows the prevalence of T2D and/or HTN by occupational activities. Most T2D sufferers are engaged in occupation that requires light or moderate activity. The respondents whose occupation requires light activity suffer most from HTN and a combination of both HTN and T2D.

## 4. Discussion

Hypertension (HTN) and Type 2 diabetes (T2D) may have an additive effect on each other, with one condition both facilitating the onset and worsening manifestations of the other (Lonati, Morganti, Comarella, Mancia, & Zanchetti, 2008). Socio-economic status (SES) and lifestyle are major determining factors with respect to the prevalence of HTN and T2D in our modern day society (Lonati et al., 2008). This study is in conformity with current body of information that a relationship between SES and T2D and/or HTN exists (Zhang et al., 2013; Corsi & Subramnian, 2012).

Fruits and vegetables are known to be very rich sources of exogenous antioxidant such as vitamins C and E. In this study, it was observed that the prevalence rate of the diseases (HTN and/or T2D) was low among those who consume fruits and vegetables daily. Our finding that more females than males suffer from diabetes may be due to the fact that most times in our society women are simply expected to be house wives who simply cook, nurture babies and satisfy the husband sexually.

It was also observed that the prevalence of T2D was more among the uneducated subjects when compared to their educated counterpart. Hence, the lower percentage rate of the disease among the postsecondary school leavers suggests better awareness and management of the disease conditions. This report is in agreement with the work of Aksu, Pala and Aksu (2006), Rajput et al. (2012) and Zhang et al. (2013). Although the prevalence rate of T2D was found to be high among those who had little or no formal education, this could possibly be due to their ignorance of the importance of fruits and vegetables among the studied groups. This was in agreement with a related study in Tianji-China which stated that education may play a role in glycaemic control among patients with T2D (Zhang et al., 2013).

It was observed that the prevalence of the diseases increases with increase in income. This could be due to the affordability and purchasing power of calorie rich diet by individuals who earn high income hence they are more prone to the disease due to cholesterol derived from consumption of junk foods. In high-income countries, the SES–T2D relationship appeared to be negative, with the poor at greatest risk (Denis et al., 2011; Dinca-Panaitescu et al., 2011; Zhang et al., 2013). According to IDF, 80% of people with T2D live in low- and middle-income countries (IDF, 2013) and indeed most Nigerians are either low or middle income earners.

The nature of occupation could also be a factor responsible for the disease prevalence as observed in this study. It was observed that those engaged in heavy occupational activities had the lowest prevalence of the disease compared with those who engage in light occupational activity. The sedentary lifestyle experienced by those who engage in light occupational activities as observed mostly among the chief executives, bankers, office secretaries and directors in the study population possibly is one of the reasons for the high prevalence of the diseases among those of light occupational activities. This observation was in harmony with the findings of other researchers that occupational activity is associated with the development of T2D seen among adults with and without HTN (United State Department of Health and Human Services [USDHHS], 2008; Aksu et al., 2006; Zhang et al., 2013; Olah et al., 2013; Xu et al., 2014). This was in agreement with a related study in Tianji-China which stated that occupational activity may play a role in glycaemic control among patients with T2D (Zhang et al., 2013).

## 5. Conclusion

From the foregoing, therefore, the prevalence of HTN and T2D can be said to be SES dependent. Therefore, we can conclude that HTN and T2D are consequences of the level of the socioeconomic state of the individual. Hence, this association between T2D and the development of HTN should prompt research on shared risk factors and alert clinicians that there is an easily identified group at high risk of HTN and T2D. Also, these information will be found useful in planning intervention healthcare preventive programs especially on public enlightenment workshops and seminars to educate people globally on the importance of lifestyle modification, healthy diet and regular exercises.
